# Psychological states affecting initial pupil size changes after olfactory stimulation in healthy participants

**DOI:** 10.1038/s41598-023-43004-1

**Published:** 2023-09-25

**Authors:** Hiroatsu Hatsukawa, Masaaki Ishikawa

**Affiliations:** Department of Otolaryngology, Head and Neck Surgery, Hyogo Prefectural Amagasaki General Medical Center, 2-17-77 Higashinaniwachou, Amagasaki, Hyogo Prefecture 660-8550 Japan

**Keywords:** Neurophysiology, Neuronal physiology

## Abstract

Odor perception affects physiological and psychological states. Pupillary light reflex (PLR) parameters can be affected by olfactory stimulation and psychological states, although it remains unclear whether the olfactory stimulation-induced psychological changes can associate with PLR parameter changes. This study aimed to investigate effects of olfactory stimulation-induced psychological changes on PLR parameter changes with repeated measurements. We collected data on six mood subscales of the profile of mood states, and on five PLR parameter measurements from 28 healthy participants. Participants underwent a 10-min olfactory stimulation on different days with six odorants available with the T&T olfactometer. As obtained data were clustered, we used linear mixed-effects models for statistical analyses. The olfactory stimulation using the no-odor liquid did not affect mood states and the initial pupil size (INIT). The sweat odorant worsened all mood subscales including fatigue–inertia (Fatigue)/Vigor–Activity (Vigor), and decreased INIT compared to the no-odor liquid. When comparing INIT responses related to changes in mood subscales between the no-odor liquid and the sweat odorant, worsened states of Fatigue/Vigor were associated with decreased INIT in the sweat odorant. Fatigue/Vigor can be used as mental fatigue indicators. Thus, mental fatigue can be associated with decreased INIT in the olfactory stimulation.

## Introduction

The odor perception affects physiological and psychological states^1^, while olfaction can be affected by aging, smoking, high body weight, and central nervous system disorders^2^. Humans can discriminate > 10^12^ olfactory stimuli^3^, due to the variety of odorant receptors binding to different odorants with unique affinity profiles^4^. These odorant receptors are expressed by olfactory sensory neurons in the olfactory epithelium. Information from activated distinct subsets of olfactory sensory neurons due to different odorants is transmitted from the olfactory bulb to the primary olfactory cortex in the piriform cortex^5^. One clinical study investigated the connectivity between the functional and structural maps of the olfactory cortex networks in normosmic participants^6^. The study revealed that the amygdala can be one of main regions associated with functional olfactory cortex maps. The amygdala is one of the common regions in emotional and olfactory processing^7^, and a part of it receives direct input from the olfactory bulb, defined as the part of the main olfactory cortex networks^8^. The three subregions (the medial amygdala, cortical amygdala, and periamygdaloid complex) in the amygdala may have distinct olfactory functions^9^. In addition, the amygdala can be associated with hedonic valence and emotional behavior in the olfactory stimulation^10,11^. The amygdala plays a key role in olfaction and other activities, such as learning and neuroendocrine stimulation modulating feeding behavior^1,7^.

The amygdala affects the autonomic mechanism controlling the pupillary light reflex (PLR)^12^. The PLR is a pupil response to light stimulation: the pupil constricts upon transient light stimulation and then gradually recovers to baseline size. In the PLR, the response is controlled by the activity of the autonomic nervous system (ANS) and is mediated by the subcortical and cortical pathways^13^. Sympathetic neurons innervate preganglionic neurons in the superior cervical ganglion through the intermediolateral column in the spinal cord, while parasympathetic neurons innervate those in the Edinger–Westphal nucleus (EWN) in the brainstem. Once light stimulus occurs pupil constriction due to the activation of the parasympathetically innervated pupillary sphincter muscle through the EWN. On the other hand, pupillary dilation occurs due to the sympathetically innervated radical dilator muscle through the intermediolateral column and the superior cervical ganglion. Stressful stimulation such as conditioned fear can activate inhibitory parasympathetic neurons in locus coeruleus (LC) via the amygdala^12,14^. As a result, the noradrenergic inhibition of the EWN can be enhanced, resulting in the inhibition of the PLR. The concept that amygdala-related stimulation can affect the PLR indicates the close association between PLR and olfactory stimulation.

The association between pupil dilation/constriction and emotional processing^13^ and between olfactory stimulation and emotion^1,7^ involves cortical modulation, including the amygdala. These findings suggest complicated relationships among odorant perception, psychological states, and PLR parameters. A recent study reviewed behavior, cognitive processes, and brain areas that modulate PLR^13^. As a result, only two studies investigated the pupillary changes due to olfactory stimulation in healthy participants^15,16^. One study investigated whether PLR parameters to olfactory stimulation could reflect their intensity/hedonic tone^15^. As a result, they reported increased initial pupil size (INIT) due to the olfactory stimulation/shortened constriction latency (LAT) due to the increased odorant intensity/no correlation between hedonic ratings and pupillary changes. They concluded that pupillary changes could reflect differences between stimulus modalities and stimulus strength, but not hedonic tone to odorants. Another study quantified pupillary and ocular responses during the simultaneous olfactory and visual stimulation using eye tracking system^16^. In addition, they investigated the association of odor recognition and the direction of attention to a picture corresponding to the odor and increased INIT. As a result, they reported increased INIT due to olfactory stimulation and gaze focus on the picture corresponding to the odor presentation. However, these studies did not investigate the effects of psychological changes induced by olfactory stimulation on pupillary states. Therefore, the association between pupillary changes and psychological changes caused by olfactory stimulation remains unclear.

We hypothesized that psychological changes due to the olfactory stimulation would be associated with changes in PLR parameters. We used five PLR parameters, the six mood subscales in the profile of mood states (POMS), and six odorants (five odorants at their highest concentration and the no-odor liquid as the control) available with the T&T olfactometer. Under a study design with repeated measurements, we assessed the following items: Effects of olfactory stimulation with the no-odor liquid on mood states (Aim 1–1) and PLR parameters (Aim 1–2); (Aim 2–1) Comparison of mood state changes at the post-olfactory stimulation among six odorants; (Aim 2–2) Comparison of PLR parameters’ changes at the post-olfactory stimulation among six odorants; (Aim 2–3) Association of mood state changes with PLR parameters’ changes at the post-olfactory stimulation.

## Materials and methods

The Research Ethics Committee of Amagasaki Medical Center approved the study protocol (approval no. 2-183). All experiments were performed in accordance with the Declaration of Helsinki. Written informed consent was obtained from all participants.

The participants working as nurses or clerks in our institution were recruited from December 2020 to March 2021, and underwent nasal endoscopy as a physical examination and a T&T olfactometer test to assess for olfactory dysfunction. Using endoscopy, nasal and sinonasal findings were used to verify that there were no anatomical structures causing nasal obstruction, such as nasal polyps, obvious septal deviation, or turbinate enlargement^2^. Participants of < 60 years of age, of healthy weight, and without central nervous disorders or mental illnesses, including Alzheimer’s and Parkinson’s diseases, autism spectrum disorder, schizophrenia, and depression were selected. A body mass index of ≥ 18.5 kg/m^2^ but < 25.0 kg/m^2^ was defined as a heaIthy weight. The Brinkman index was used to assess the intensity of smoking, calculated as the number of cigarettes smoked per day multiplied by the number of years of smoking.

The T&T olfactometer test (Daiich Yakuhin Sangyo, Tokyo, Japan) consists of five odorants and a no-odor liquid control—paraffin. The names of the odors are as follows: (A) roses (β-phenylethyl alcohol), (B) burning (methyl cyclopentenolone), (C) sweat (isovaleric acid), (D) fruits (γ-undecalactone), and (E) vegetable chips (skatol). Seven concentrations were available in log_10_ serial dilutions (from − 2 to 4) for methyl cyclopentenolone, while eight were available for the other odorants (from − 2 to 5). Average scores for the recognition of the five odorants were calculated, and participants with scores of < 1.1 were defined as individuals without olfactory loss^17^. For these participants, the highest concentration of each odorant was used for olfactory stimulation in subsequent tests.

In total, 28 participants were recruited. The T&T olfactometer showed average scores (for recognition of five odorants) of < 1.1, and the nasal endoscopy showed no anatomical obstructions. The median age (IQR), male ratio and Brinkman index were 29 (26–40), 50% and 0 (0–16), respectively.

The POMS 2nd edition–adult short version was used^18^. Scores for the following six mood subscales, ranging from 0 to 4, were determined: Anger–Hostility (Anger); Confusion–Bewilderment (Confusion); Depression–Dejection (Depression); Fatigue–Inertia (Fatigue); Tension–Anxiety (Tension); Vigor–Activity (Vigor). Each score was converted to standardized scores and adjusted based on age and sex^18^. As the interpretation of mood subscale changes at post olfactory stimulation, increased scores in all mood subscales except Vigor indicated worsened states, while for Vigor, decreased scores indicated a worsened state.

**Figure 1:**
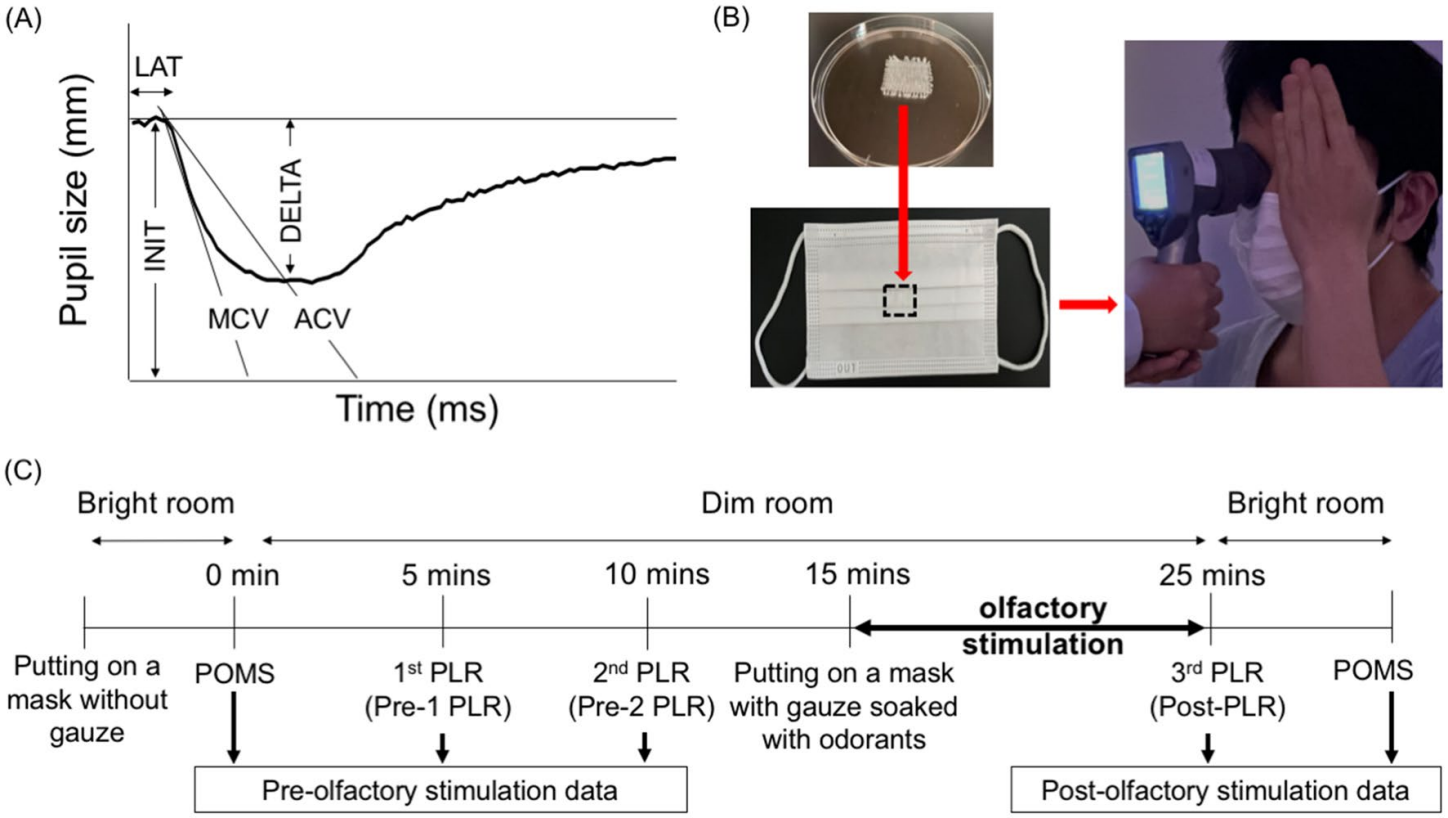
PLR parameters adopted, olfactory stimulation using a facemask, and study design. (**A**) Five PLR parameters were used for statistical analyses: ACV, DELTA, INIT, LAT, and MCV. (**B**) A 2 × 2 cm sterile gauze was soaked in an odorant in a petri dish. The soaked sterile gauze (dotted square) was sandwiched between folds of a commercially available facemask. The participant was asked to put on the mask so that the side sandwiching the gauze was on the face side. In a dim room, PLR measurement was performed using a commercially available pupillometer. To prevent stimulation by ambient light to the eyes, the sides of both eyes were covered with the participant’s hand or the rubber cup of the pupillometer. (**C**) The time schedule and the collected data are shown. For each time point of PLR measurement, eight data points (2 eye sides × 4 light stimulus intensities) were collected for each participant. The 1st and 2nd PLR data were defined as pre-1 PLR and pre-2 PLR, respectively. The 3rd PLR data were defined as post-PLR. *ACV* averaged constriction velocity, *DELTA* constriction ratio, *INIT* initial pupil size, *LAT* constriction latency, *MCV* maximum constriction velocity, *PLR* pupillary light reflex, *POMS* profile of mood states.

The handheld infrared pupilometer (PLR-3000; NeurOptics, Irvine, CA) used in this study was equipped with a rubber cup to cover the measured eye. It provided measurements of six PLR parameters, and continuous values automatically: INIT, constriction ratio (DELTA), LAT, average constriction velocity (ACV), maximum constriction velocity (MCV), and average dilation velocity (T75). As the average dilation velocity and T75 were not measurable when participants blinked during the PLR measurement, five PLR parameters (ACV, DELTA, INIT, LAT, and MCV) were used for the analyses (Fig. 1A). As light stimulus intensities affect PLR parameters^19,20^, four different intensities (10, 50, 121, and 180 μW) were applied for PLR. The recording duration was 5 s with 0 μW of background intensity. For PLR measurements using different intensities, a step-up method was adopted in the same PLR setting (see Supplementary Fig. 1), as previously reported^19–22^. The order of the measurement was 10 μW right eye, 10 μW left eye, 50 μW right eye, 50 μW left eye, 121 μW right eye, 121 μW left eye, 180 μW right eye, 180 μW left eye. The intervals between measurements lasted 30 s.

### Study design

A 2 × 2 cm sterile gauze (HM-1301, HOGY Medical. Co Ltd.) was placed on a Petri dish (AB 4000, Eiken Chemical Co Ltd.) containing 2 mL of an odorant selected from the T&T olfactometer test (Fig. 1B). The sterile gauze soaked in the odorant was then sandwiched between the folds of a commercially available face mask (Pro Care, A.R. Medicom. Inc. (Asia) Ltd.). For PLR measurements, participants were asked to wear a face mask. The unmeasured eye was then covered with their hand to prevent ambient light stimulation^23,24^, while the measured eye was covered with a rubber cup of the pupilometer. PLR measurements were performed after maximizing the pupil size by covering both eyes.

The study design is illustrated in Fig. 1C. In the bright room, participants were asked to put on a mask without a gauze sample and then fill out the POMS questionnaire while seated. The first and second PLR measurements were performed 5 and 10 min after dimming the lights in the room, respectively. These POMS- and PLR-related data were considered pre-olfactory stimulation data. The PLR data obtained at the first and second PLR measurements were defined as pre-1 PLR and pre-2 PLR, respectively. Fifteen minutes after dimming the lights in the room, they were asked to put on a mask with gauze soaked in the selected odorant. During olfactory stimulation, participants were asked to remain silently seated. After 10-min olfactory stimulations, a third PLR measurement was performed. For each PLR measurement, eight data points were collected from both eyes at 10, 50, 121, and 180 μW. Finally, they were asked to complete the questionnaire again in a brightly lit room. These POMS- and PLR-related data were regarded as post-olfactory stimulation data. The PLR data at the third PLR measurement were defined as post-PLR data. All experiments were conducted at 4–7 pm on different days, within 14 days, to minimize the effects of variability between and within participants on the PLR parameters. A single measurer was put on an N-95 nano mask (HI-LUCK, KOKEN, Japan) to avoid the detection of the odorant and then performed all PLR measurements. An assistant performed a random selection of odorants, without informing either the measurer or the participants. Therefore, our study design was double-blinded with repeated measurements.

### Data analysis

Quantitative variables are represented as medians or means with interquartile ranges or 95% confidence intervals (CI).

For the aim 1–1, the Wilcoxon signed-rank test was used to assess changes in mood subscales between the two time-points. Commercially available software (GraphPad Prism, GraphPad Software Inc., La Jolla, CA, USA) was used for all analyses.

For aims 1–2, 2–1, 2–2, and 2–3, the data set could be regarded as one that includes cluster information with non-independent observational units, due to the repeated measurements^25^. For instance, for aims 1–2, PLR data were collected at three time-points under four different light stimulus intensities bilaterally; for aims 2–1, 2–2, and 2–3, all values were obtained from participants using different odorants on different days. Therefore, the data can be regarded as including individual variabilities^26^. Adjusting the variability could help reduce false-positive and -negative ratios^27^, which requires an appropriate statistical method. Linear mixed-effects models (LMMs) consist of fixed and random effects, and individual variability can be adjusted using random effects. Therefore, LMMs may be one of the appropriate statistical methods for analyzing data that include cluster information^25^. Therefore, we used LMMs in commercially available software (version 3.6.1; R Foundation for Statistical Computing, Vienna, Austria; lme4 and lmerTest packages). In LMMs, the data were regarded as normally distributed according to the central limit theorem^28^.

For aims 2–1, 2–2 and 2–3, we calculated changes (Δ) of mood subscales/PLR parameters by subtracting the values at pre-olfactory stimulation from those at post-olfactory stimulation. The sample sizes of ΔPLR parameters were bigger than those of Δmood subscales because the PLR data were collected by the more complicated conditions: The POMS data were collected at two time-points, while the PLR data were collected by three time-points, from bilateral eye sides, and at four different light stimulus intensities. For instance, when analyzing INIT collected by right side at 180 μW, both of the following two values were used for analyses: (1) post-INIT–pre-1 INIT; (2) post-INIT–pre-2 INIT. For the aim 2–3, Δmood subscales × odorant selection were added in LMMs.

The age, the Brinkman index, and the intensity of the light stimulus, and mood subscales/PLR parameters-related values were defined as continuous variables, while sex and measured eye side (right/left) as dichotomous variables. A measured time-point was defined as categorical variable, and then pre1-PLR data was used as a reference. The odorant selection for aims 2–1 and 2–2 was defined as categorical variables, one for aim 2–3 as a dichotomous variable. In all aims, the no-odor liquid was used as a reference. Each participant identification number (n = 28 participants) was defined as a random effect to adjust for individual variability.

The following formula of LMMs were used in the software of R:

$$\begin{aligned} {\text{For aim 1}} {-} 2{:}\;{\text{PLR parameters}} & = {\text{lmer}}\;({\text{intercept}} + {\text{measured time point}} + {\text{age}} + {\text{sex}} \\ & \quad + {\text{Brinkman index}} + {\text{measured eye sides}} + {\text{light stimulus intensity}} \\ & \quad + (1|{\text{participantidentification number}})). \\ \end{aligned}$$

$$\begin{aligned} {\text{For aim 2}} {-} 1{:}\;\Delta {\text{a targeted mood subscale}} & = {\text{lmer}}({\text{Intercept}} + {\text{age}} + {\text{sex}} + {\text{Brinkman index}} + {\text{odorant selection}} \\ & \quad + {\text{baseline values of a targeted mood subscale at pre-olfactory stimulation}} \\ & \quad + (1|{\text{participantidentification number}})). \\ \end{aligned}$$

$$\begin{aligned} {\text{For aim 2}} {-} {2:}\;\Delta {\text{a targeted PLR parameters}} & = {\text{lmer}}\;({\text{intercept}} + {\text{odorant selection}} + {\text{age}} + {\text{sex}} + {\text{Brinkman index}} \\ & \quad + {\text{measured eye sides}} + {\text{light stimulus intensity}} + \Delta {\text{Anger}} + \Delta {\text{Confusion}} \\ & \quad + \Delta {\text{Depression}} + \Delta {\text{Fatigue}} + \Delta {\text{Tension}} + \Delta {\text{Vigor}} \\ & \quad + {\text{baseline values of a targeted PLR parameter at the pre-olfactory stimulation}} \\ & \quad + (1|{\text{participantidentification number}})). \\ \end{aligned}$$

$$\begin{aligned} {\text{For aim 2}} {-} {2:}\;\Delta {\text{a targeted PLR parameters}} & = {\text{lmer}}\;({\text{intercept}} + {\text{odorant selection}} + {\text{age}} + {\text{sex}} + {\text{Brinkman index}} \\ & \quad + {\text{measured eye sides}} + {\text{light stimulus intensity}} + \Delta {\text{Anger}} \\ & \quad + \Delta {\text{Confusion}} + \Delta {\text{Depression}} + \Delta {\text{Fatigue}} + \Delta {\text{Tension}} + \Delta {\text{Vigor}} \\ & \quad + {\text{baseline values of a targeted PLR parameter at the pre-olfactory stimulation}} \\ & \quad + (1|{\text{participantidentification number}})). \\ \end{aligned}$$

$$\begin{aligned} {\text{For aims 2}} {-} {3:}\;\Delta {\text{INIT}} & = {\text{lmer}}\;\left( {{\text{intercept}} + {\text{odorant selection}} \times \Delta {\text{Anger}} + {\text{odorant selection}}} \right. \\ & \quad \times \Delta {\text{Confusion}} + {\text{odorant selection}} \times \Delta {\text{Depression}} + {\text{odorant selection}} \\ & \quad \times \Delta {\text{Fatigue}} + {\text{odorant selection}} \times \Delta {\text{Tension}} + {\text{odorant selection}} \\ & \quad \times \Delta {\text{Vigor}} + {\text{age}} + {\text{sex}} + {\text{Brinkman index}} + {\text{measured eye sides}} \\ & \quad + {\text{light stimulus intensity}} + {\text{baseline values of INIT at the pre-olfactory stimulation}} \\ & \quad \left. { + \left( {1|{\text{participant identification number}}} \right)} \right) \\ \end{aligned}$$

In all analyses, *p* values < 0.05 were considered statistically significant differences .

## Results

### Aim 1–1: effects of olfactory stimulation with the no-odor liquid on mood states

**Figure 2:**
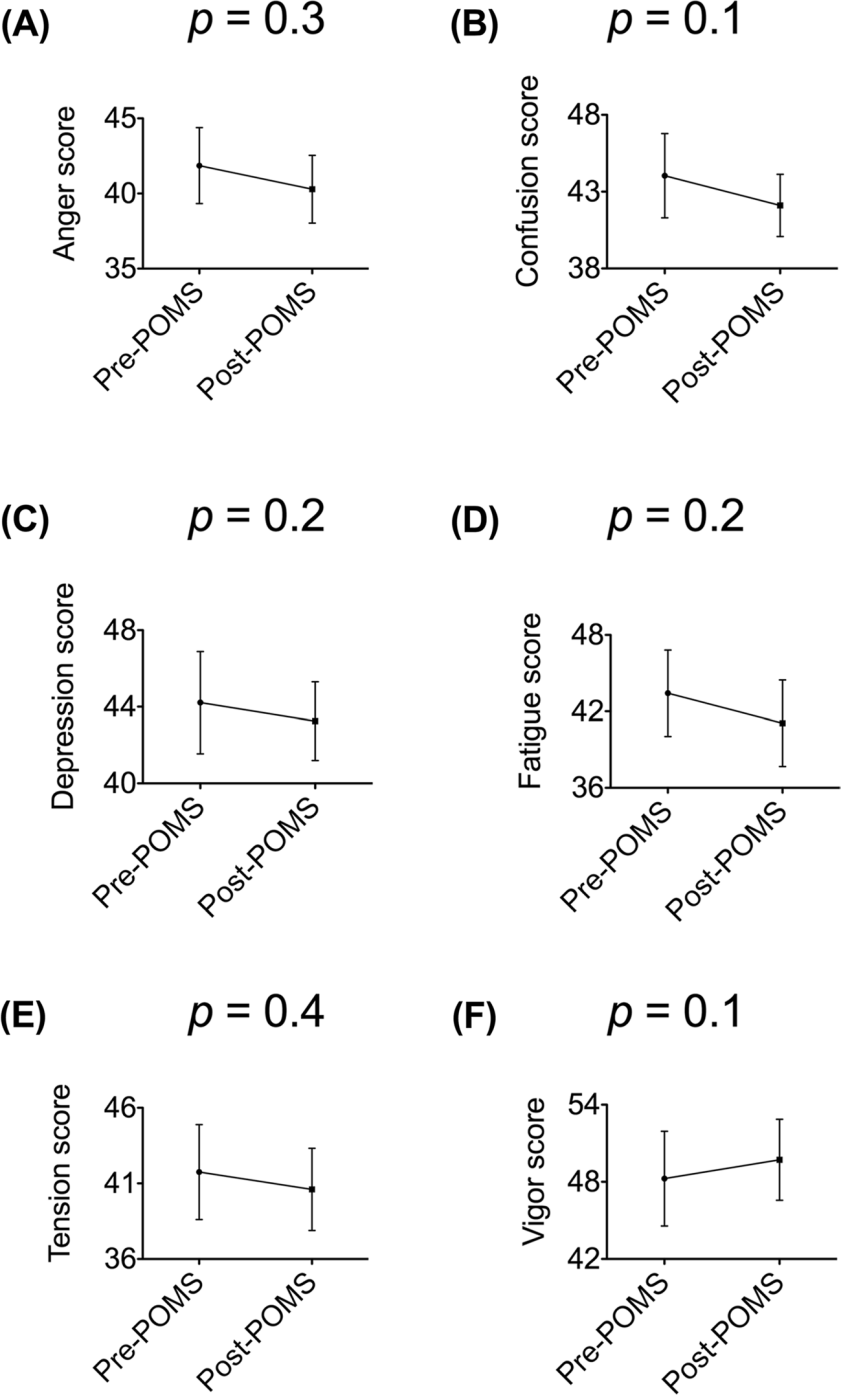
Time-course changes in six mood subscale scores with olfactory stimulation using the no-odor liquid. (**A**) Anger, (**B**) Confusion, (**C**) Depression, (**D**) Fatigue, (**E**) Tension, and (**F**) Vigor. All values represent means and 95% confidence intervals (n = 56 data points: 28 participants × 2 time-points). A Wilcoxon signed-rank test is used for statistical analyses. *Anger* Anger–Hostility, *Confusion* confusion–bewilderment, *Depression* depression–dejection, *Fatigue* fatigue–inertia, *POMS* profile of mood state, *Tension* tension–anxiety, *Vigor* Vigor–Activity.

First, to investigate the psychological effects of olfactory stimulation using the no-odor liquid, we compared the time-course changes in six mood subscales. Fifty-six data points (28 participants × 2 time-points) for each POMS subscale were used for statistical analyses to each mood subscale. As data were not clustered, we used the Wilcoxon signed-rank test for statistical analyses. There were no significant differences in the six mood subscales between pre- and post-olfactory stimulation time-points (Fig. 2).

### Aim 1–2: effects of olfactory stimulation with the no-odor liquid on PLR parameters

**Figure 3:**
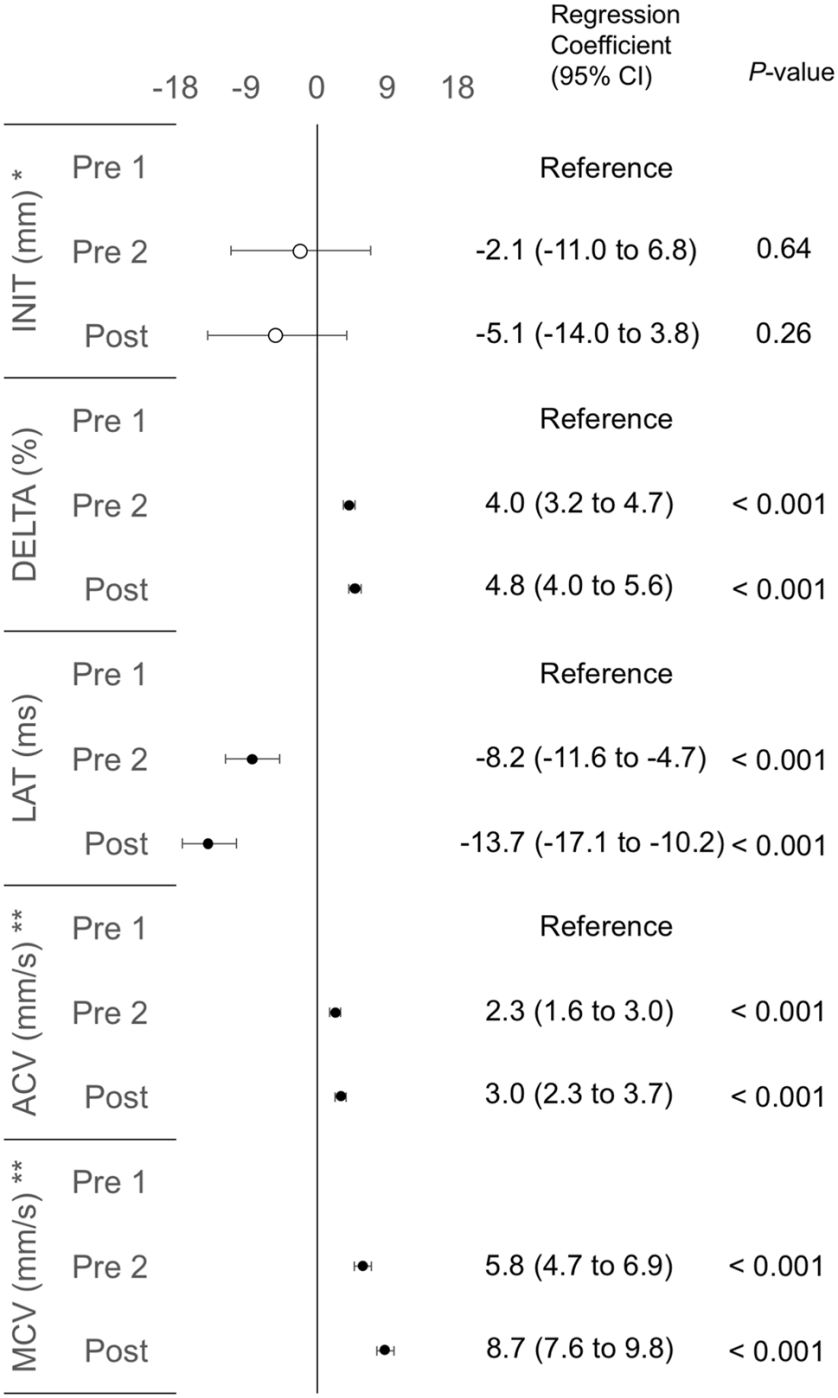
Forest plot of regression coefficients showing time course changes of PLR parameters in the olfactory stimulation using the no-odor liquid. The estimated regression coefficients are obtained by linear mixed-effects models. Black circles indicate significant regression coefficients. Pre-1 PLR data are set as references. *regression coefficient × 10^−2^; **regression coefficient × 10^−1^. *CI* confidence interval, *ACV* averaged constriction velocity, *LAT* constriction latency, *DELTA* constriction ratio, *INIT* initial pupil size, *MCV* maximum constriction velocity, *PLR* pupillary light reflex, *Pre 1, Pre 2, and Post* pre-olfactory stimulation at 1st, 2nd, and post-olfactory stimulation, respectively.

Next, we investigated whether olfactory stimulation using no-odor liquid can affect PLR parameters. In total, 672 data points (28 participants × 4 light stimulus intensities × 2 measured eye sides × 3 time-points) were used for statistical analyses. As the obtained data were clustered, we used LMMs and then evaluated time course changes of PLR parameters based on the regression coefficients (Fig. 3). Compared with pre1-PLR data, pre2-PLR data showed significant regression coefficients in DELTA, LAT, ACV, and MCV but not INIT: Increased DELTA, ACV, and MCV, and shortened LAT. The same findings were observed when comparing between pre1- and post-PLR data.

Taken together, in the no-odor liquid, INIT was the only parameter not affected at the pre- and post-olfactory stimulation time-points.

### Aim 2–1: comparison of mood state changes at the post-olfactory stimulation among six odorants

**Figure 4:**
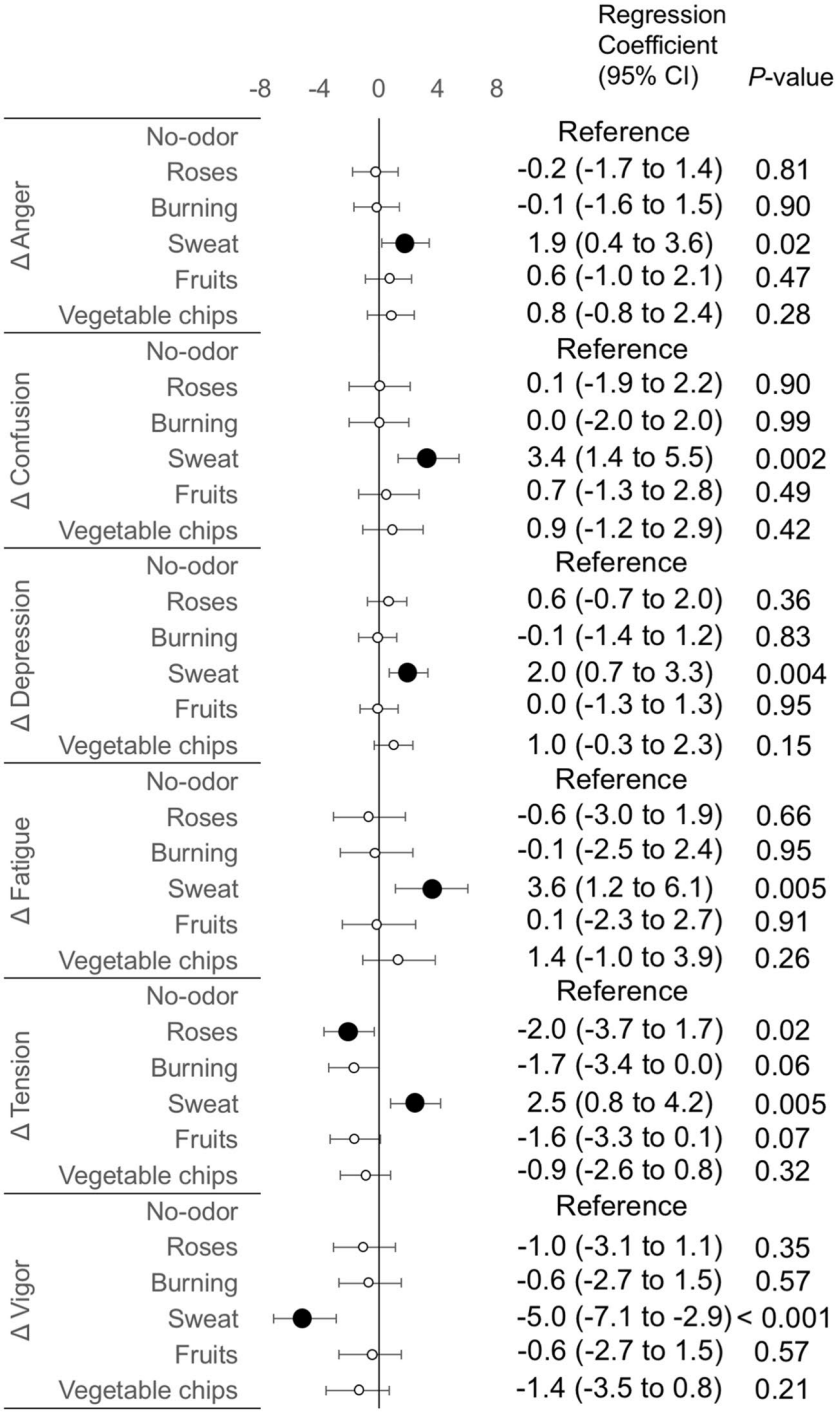
Forest plot of regression coefficients showing changes of mood subscales at the post-olfactory stimulation using six odorants. For the six odorants, changes in mood subscales calculated by subtracting values at pre-olfactory stimulation from those at post-olfactory stimulation are shown by Δ. Estimated regression coefficients and *p* values calculated by linear mixed-effects models are shown. The no-odor liquid is set as a reference. Black circles indicate significant regression coefficients. *Anger* Anger–Hostility, *CI* confidence intervals, *Confusion* confusion–bewilderment, *Depression* depression–dejection, *Fatigue* fatigue–inertia, *Tension* tension–anxiety, *Vigor* Vigor–Activity.

We investigated which odorants can change psychological states at the post-olfactory stimulation. In total, 168 data points (28 participants × 6 odorants) were used for statistical analyses. In LMMs, compared to the no-odor liquid, the sweat odorant showed significant regression coefficients in all mood subscales: increased Anger, Confusion, Depression, Fatigue, and Tension, and decreased Vigor (Fig. 4). Thus, the sweat odorant markedly worsened all mood states. However, the effects were not universal in all participants. The scatter plots showing Δmood subscales at the post-olfactory stimulation were shown in supplementary Fig. 2. For example, some participants showed below zero values for ΔFatigue (supplementary Fig. 2D), indicating they had not experienced worsened Fatigue after olfactory stimulation with the sweat odorant.

### Aim 2–2: comparison of PLR parameters’ changes at the post-olfactory stimulation among six odorants

**Figure 5:**
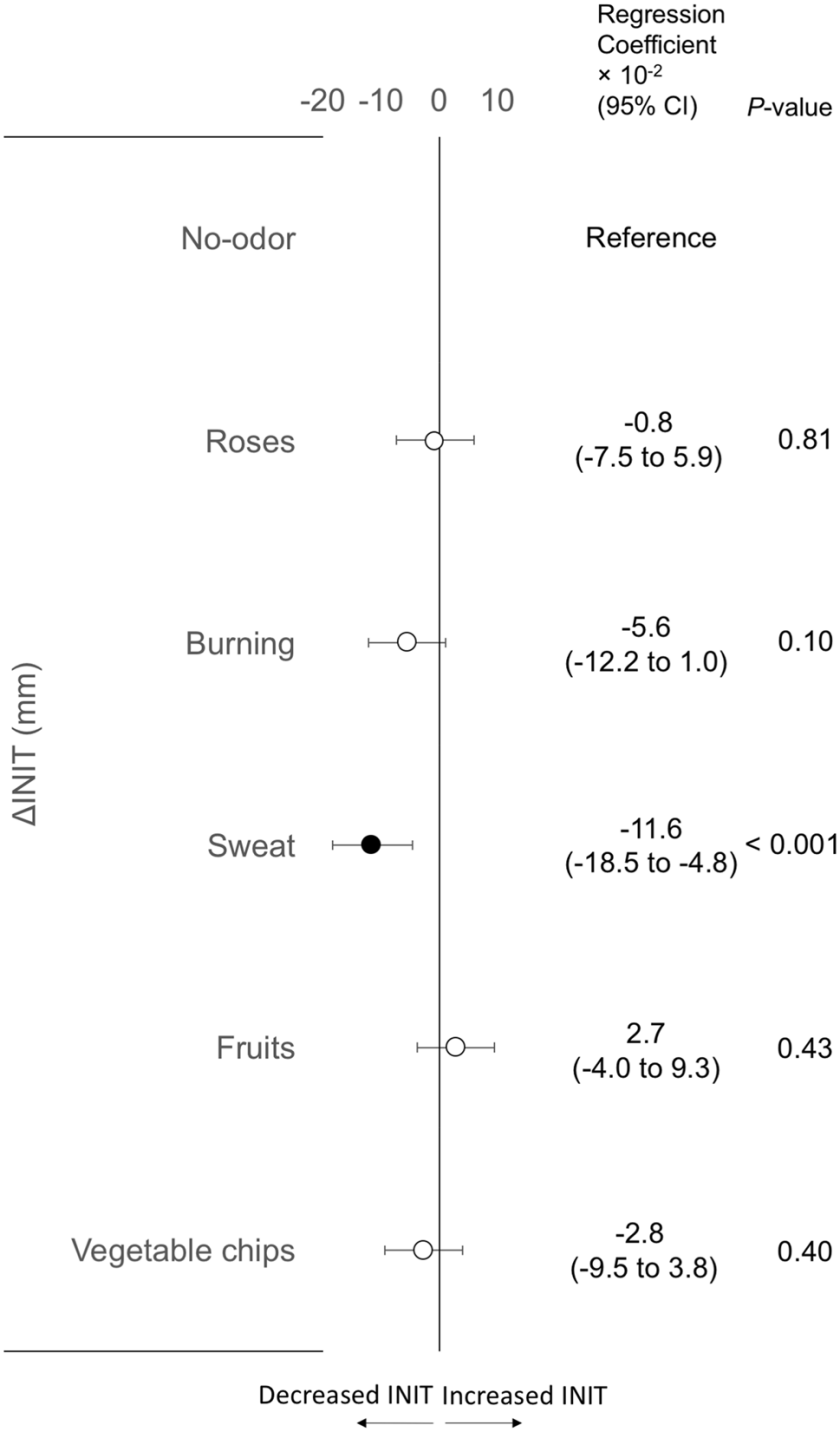
Forest plot of regression coefficients showing changes of INIT at the post-olfactory stimulation among six odorants. For each of six odorants, INIT changes are calculated by subtracting values at pre-olfactory stimulation from those at post-olfactory stimulation, shown by Δ. Regression coefficients and *p* values calculated by linear mixed-effects models are shown. The no-odor liquid is set as a reference. *CI* confidence interval, *INIT* initial pupil size.

Next, we investigated the effect of the five odorants (roses, burning, sweat, fruits, and vegetable chips) on all PLR parameters, compared to the no-odor liquid. Among a total of 2688 data points (28 participants × 2 sides × 4 different intensities of light stimulus intensities × 2 time-points × 6 odorants), regression coefficients estimating ΔPLR parameters at the post-olfactory stimulation were calculated by LMMs. Compared to the no-odor liquid, sweat odorant showed a significant regression coefficient: decreased INIT (Fig. 5). In other PLR parameters, significant regression coefficients were not observed for all odorants (Supplementary Fig. 3).

### Aim 2–3: association of mood state changes with PLR parameters’ changes at the post-olfactory stimulation

**Figure 6:**
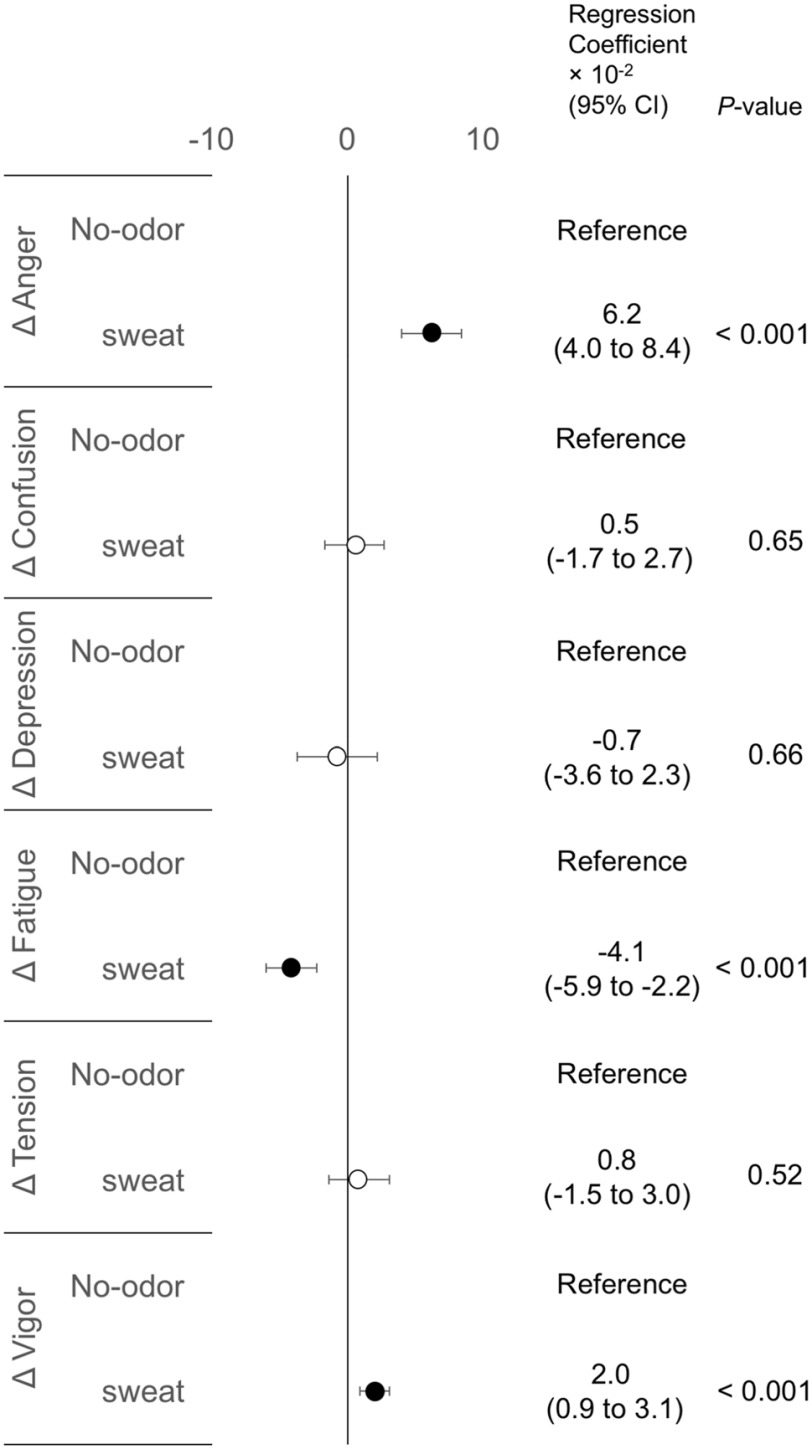
Forest plot of regression coefficients showing initial pupil size responses due to mood subscale changes at the post-olfactory stimulation using the no-odor liquid and the sweat odorant. The change values calculated by subtracting values at pre-olfactory stimulation from those at post-olfactory stimulation are shown by Δ. Regression coefficients and *p* values calculated by linear mixed-effects models are shown. The no-odor liquid is set as a reference. *Anger* Anger–Hostility, *CI* confidence interval, *Confusion* confusion–bewilderment, *Depression* depression–dejection, *Fatigue* fatigue–inertia, *Tension* tension–anxiety, *Vigor* Vigor–Activity.

**Figure 7:**
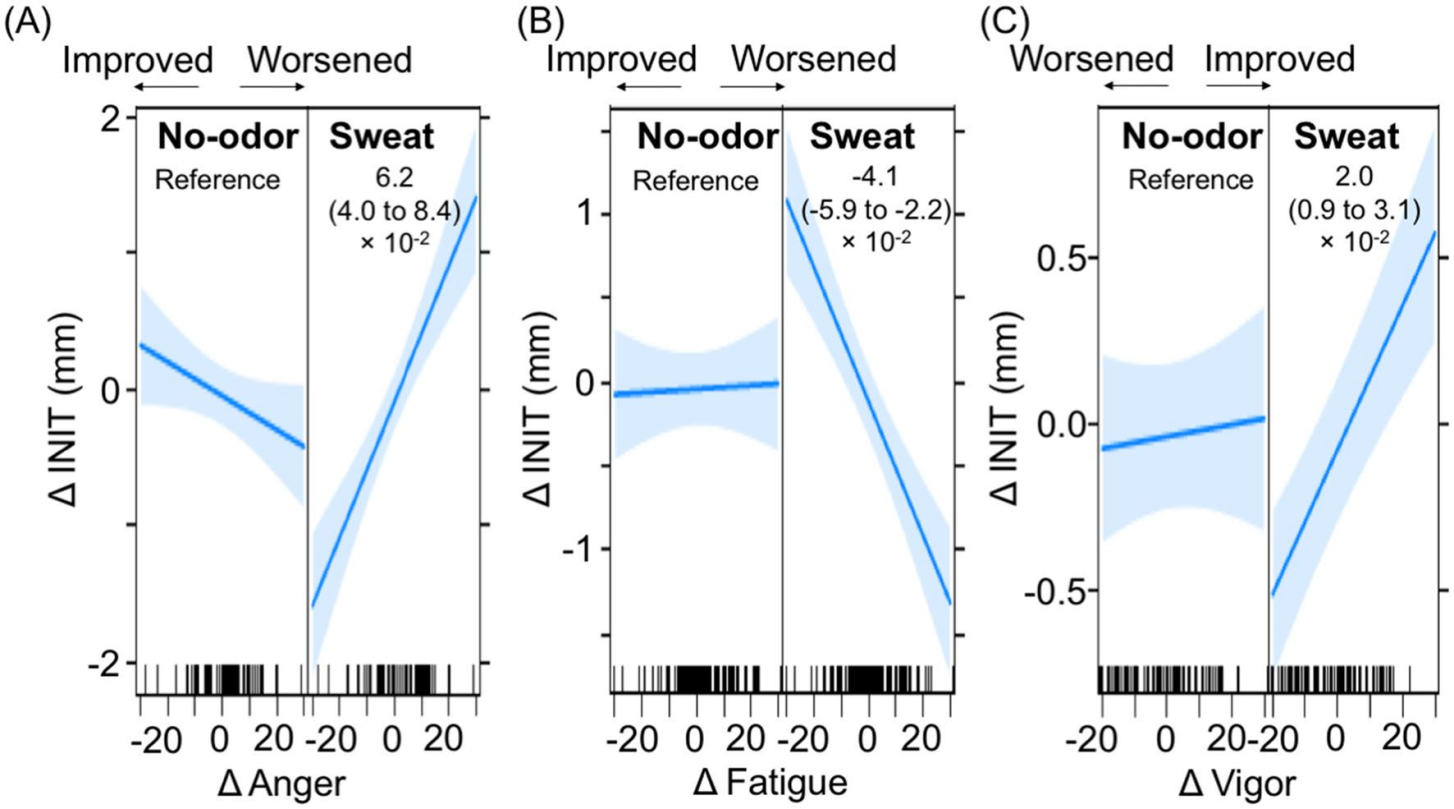
Comparison of initial pupil size responses due to three mood subscales’ changes between the no-odor liquid and the sweat odorant. The change values calculated by subtracting values at pre-olfactory stimulation from those at post-olfactory stimulation are shown by Δ. The graphs show the slopes reflecting the mean ΔINIT to Δmood subscales with 95% confidence intervals: (**A**) ΔAnger, (**B**) ΔFatigue, and (**C**) ΔVigor. Regression coefficient values of interaction effects calculated using linear mixed-effects models are shown in each figure. The interaction effect represents the differences of slopes between the no-odor liquid and the sweat odorant. The no-odor liquid is set as a reference. *Anger* Anger–Hostility, *Fatigue* fatigue–inertia, *INIT* initial pupil size, *Vigor* Vigor–Activity.

As the olfactory stimulation using sweat odorant worsened all mood subscales (Fig. 4) and decreased INIT (Fig. 5), we considered that a comparison between the no-odor liquid and sweat odorant might help identify the mood subscale most involved in decreased INIT. We used data from the no-odor liquid and sweat odorant and then compared the slopes showing the association between ΔINIT and Δmood subscales. In total, 896 data points (28 participants × 2 sides × 4 different light stimulus intensities × 2 time-points × 2 odorants) were used in LMMs. The significant regression coefficient of interaction effects (odorant selection × Δmood subscales) indicates that INIT responses due to mood subscale changes can differ between the two odorants. As a result, in comparison to the no-odor liquid, the sweat odorant showed significant regression coefficients in Anger, Fatigue, and Vigor: positive values of Anger and Vigor; negative values of Fatigue (Fig. 6). Focused on these three mood subscales, we illustrated slope differences between the two odorants (Fig. 7). With the no-odor liquid, flat slopes were observed for Anger, Fatigue, and Vigor, while with the sweat odorant, there was a positive slope for Anger (Fig. 7A)/Vigor (Fig. 7C) and a negative slope for Fatigue (Fig. 7B). Taken together, the decreased INIT in the sweat odorant was associated with improved states of Anger or worsened states of Fatigue/Vigor.

## Discussion

This study investigated the association between psychological changes and PLR parameter changes in olfactory stimulation. In olfactory stimulation using the no-odor liquid, INIT was the only parameter less affected by pre- and post-olfactory stimulation time-points. When we compared changes in the mood states and PLR parameters among the six odorants, the sweat odorant worsened all mood states, including Fatigue/Vigor, and decreased INIT, compared to the no-odor liquid. In the INIT responses due to mood state changes at post-olfactory stimulation, the comparison between the no-odor liquid and the sweat odorant showed that decreased INIT in the sweat odorant can be associated with improved states of Anger or worsened states of Fatigue/Vigor.

The T&T olfactometer is the psychophysical tool assessing odor threshold and identification for the diagnosis of olfactory impairment^29^. The psychophysical tools, such as the T&T olfactometer can be more reliable assessments of olfactory function than subjective reporting, but these tools still require a cooperative participant, who understands and follows instructions and communicates choices to the clinician/investigator. The establishment of psychophysical tools, which do not require these cooperation, might improve the clinical ability to diagnose olfactory impairment. One review targeting animal models reported the change of autonomic nerve activity and physiological function in the olfactory stimulation^30^: The olfactory stimulation using different odors can affect various tissues and organs via the different modalities of the autonomic nervous system. The PLR is one of the representative tools reflecting the activity of ANS in a non-invasive way, and there were two clinical studies investigating the association between pupil features and olfactory stimulation^15,16^. Based on the research backgrounds and clinical application in the future, we combined the T&T olfactometer with PLR with the aim to establish the psychological tool enabling us objective assessment in the effects of olfactory stimulation.

To investigate whether the olfactory stimulation adopted in the current study could affect mood states and PLR parameters, we assessed the effects of olfactory stimulation using the no-odor liquid on six mood subscales of the POMS and five PLR parameters. Olfactory stimulation using the no-odor liquid did not affect mood states (Fig. 2). Theoretically, selecting PLR parameters that are less affected by factors, except mood states, would be reliable to assess the effects of mood states on PLR parameters in the olfactory stimulation. Therefore, INIT could be regarded as the most reliable parameter in our study. The intervals between the beginning of pre2-PLR and the end of the pre1-PLR were approximately 50 s (see Supplementary Fig. 1). The insufficient intervals could explain why DELTA, ACV, and MCV increased, and LAT decreased in the pre-2 PLR (see Fig. 3). One previous study showed an increased DELTA, ACV, and MCV and a shortened LAT with increased light stimulus intensities^20^. The findings of the DELTA, LAT, ACV, and MCV changes observed in the pre-2 PLR indicated the residual effects of PLR performed in the pre1-PLR. Prolonged intervals between pre-1 and pre-2 PLR might reduce parameter changes, improving the clinical potential of these PLR parameters as indicators of the psychological effects of olfactory stimulation on PLR parameters. At post-olfactory stimulation, all parameters except INIT showed significant differences despite no mood changes (see Fig. 3). However, the reason remains unclear, warranting further studies.

The use of POMS might be effective in revealing how the psychological changes due to stimulation can affect PLR parameters. Two studies using subacute pain models reported a decrease in INIT due to the increase of subjective pain intensity^21,22^, which was an unexpected finding. This is because pain stimulation can lead to pupil dilation through increased sympathetic outflow to the pupil from the LC^12,14^. To explain the finding, two studies used POMS, and then explained how the decreased INIT could occur even during the experience of pain from the point of view of psychological states^31,32^: The increase of subjective pain intensity caused the worsened states of Fatigue/Vigor, which were associated with decreased INIT. In POMS, Fatigue and Vigor can be valid indicators of mental fatigue^33^. They concluded that subacute pain-induced-mental fatigue can be associated with decreased INIT. Mental fatigue is a type of physiological fatigue due to cognitive processing for a period of time; this can reduce cognitive capacity and motivation to continue the task^34^. A systematic review stated that the decreased INIT is observed in cognitive tasks, causing mental fatigue^34^. However, inconsistent findings about the association between mental fatigue and INIT have been reported: cognitive tasks were found to be associated with the non-decreased INIT^35^, the increased INIT^36^, and decreased INIT^37^. These findings may be due to different degrees of mental fatigue resulting from cognitive tasks with different intensities, different subjective methods used to assess degrees of mental fatigue, and different environmental settings. As POMS-related scores are continuous variables, it enables us to evaluate the degree of psychological state changes quantitatively. In addition, the different stimulation effects on psychological states can be uniformly assessed. Therefore, we used POMS in the current study.

When we evaluated the association between ΔINIT and Δmood subscales in response to the six odorants, we focused on the comparison between the no-odor liquid and sweat odorant because the sweat odorant affected all mood subscales (Fig. 4) and decreased INIT (Fig. 5). We plotted ΔINIT to Δmood subscales for both odorants and compared the slopes. In the sweat odorant, the decreased INIT was associated with worsened Fatigue/Vigor and improved Anger (Fig. 7). As the sweat odorant worsened all mood subscales in POMS (Fig. 4), we speculated that the decreased INIT might be associated with worsened states of Fatigue/Vigor, rather than improved states of Anger. Participants who showed worsening states of Fatigue and Vigor due to olfactory stimulation with sweat odorant could be regarded as experiencing a state of mental fatigue.

Regarding the effects of olfactory stimulation with the six odorants on mood states, we observed changes mainly with the sweat odorant (Fig. 4). However, changes in mood states showed variability among individuals, and some participants did not show deterioration in mood subscale states (Supplementary Fig. 2). Further studies should clarify the olfactory stimulation method that induces worsening mood states more universally, particularly mental fatigue (i.e., by adjustment of intervals for olfactory stimulation).

Our current study showed that mental fatigue was the main psychological factor associating with the decreased INIT in the olfactory stimulation, but disgust also can be considered. For example, one clinical study using visual stimulation reported that the pupil reactions due to images eliciting disgust can differ from those caused by eliciting fear^38^: Greater decreased INIT was observed in images eliciting disgust than fear. The authors concluded that the visual stimulation eliciting disgust can cause decreased INIT, which can be associated with enhanced parasympathetic activity. The olfactory stimulation using the sweat odorant might cause not only mental fatigue but also disgust, which is associated with decreased INIT.

The LC-noradrenergic (LC-NE) system might explain the mechanism of the decreased INIT due to mental fatigue^35,37^. The LC-NE system consists of two different modes of attentional control: phasic and tonic modes^39–41^. The intermediate baseline levels of NE and strong stimulus-evoked bursts of NE release are observed in the phasic mode, while both the high baselines of NE and the high stimulus-evoked bursts of NE release in tonic mode^35^. The phasic mode supports high task engagement^42,43^, while the tonic mode causes decreased phasic LC activity and worsened task engagement^44,45^. The tonic mode can possess a third output mode with the low baselines of NE and the low stimulus-evoked bursts of NE release^35^, leading to diminished attention, task disengagement, and decreased vigor^44^. These behaviors can be observed in people with a state of mental fatigue^46,47^. The phasic mode drives the exploitation of the task with an aim to optimize the task rewards, while the tonic mode exploration of the environment to find more rewarding tasks^48^. The LC is the hub of the pupil-control pathway related to NE^12^. In the sympathetic outflow from the LC to the radical dilator muscle, the projections derived from the sympathetic premotor neurons in the LC are likely to be excitatory via postsynaptic α1-adrenoceptors to the intermedio-lateral column^12^. In the parasympathetic outflow from the LC to the pupillary sphincter muscle, the projections derived from the parasympathetic premotor neurons in the LC are inhibitory via postsynaptic α2-adrenoceptors to the EWN^12^. The lower output of NE from the LC in the third output mode might cause decreased sympathetic activity via the postsynaptic α1-adrenoceptors and/or increased parasympathetic activity due to insufficient inhibition of EWN. As the INIT is the consequence of balanced activity in the sympathetic and parasympathetic nervous system^49^, the decreased sympathetic activity and/or the increased parasympathetic activity via the LC-NE system might cause decreased INIT. However, the increased parasympathetic activity in the pupil due to mental fatigue remained debatable because our current study showed no change of the other parasympathetic indicators in PLR parameters even when the olfactory stimulation using the sweat odorant was conducted. One previous study using the human pupillary muscle plant model reported increased MCV due to increased parasympathetic activity^50^. As described above, increased light stimulus intensity causes the shortened LAT, increased ACV/MCV, and increased DELTA^20^. These changes might be regarded as the indicators reflecting the increased parasympathetic nervous activity. Therefore, the decreased INIT observed in our current study might be due to the decreased sympathetic activity related to mental fatigue rather than the enhanced parasympathetic activity. As the salivary gland is controlled by the parasympathetic outflow of the LC^12^, the combination of the PLR and salivary gland in the olfactory stimulation might be helpful to explain the mechanism of changed activity in ANS observed in our study.

Our study had limitations. First, we presented olfactory stimulation with each odorant on different days, which could lead to different mood states and PLR parameters, even within participants and even before olfactory stimulation. To overcome this issue, we added pre-olfactory stimulation data of POMS/PLR as explanatory variables in LMMs with the aim of adjusting baseline data. Second, the sample size of this study was small. However, to overcome this issue, we adopted a study design with repeated measurements and used LMMs to adjust individual variability. We obtained 168 data points for each mood subscale and 2688 data points for each PLR parameter from 28 participants and used these in LMMs. Third, although sleepiness/sleep loss can cause a decrease in INIT^51–53^, we did not assess the effects of olfactory stimulation on sleepiness. Recent studies have stressed the importance of differentiating sleepiness from mental fatigue^34,54^. Further studies should differentiate whether the decreased INIT observed in our current study was due to mental fatigue or sleepiness. Fifth, our study used the highest concentration of each odorant in the T&T olfactometer test and did not investigate the effects of different odorant intensity on mood states and PLR parameters. As the previous study reported changes in PLR parameters due to the increased odorant intensities^15^, it is the next issue to investigate the effects of different odorant intensities on the association between changes in mood states and PLR parameters due to the olfactory stimulation. Sixth, from viewpoints of the quantitative methodology, the olfactory stimulation using the mask should be modified. The use of air-dilution olfactometry can adjust the quantitative effects more precisely^15,55^. As our current study was conducted during the COVID-19 pandemic, and the olfactory stimulation using the air-dilution flow system was not acceptable for preventing the infection. The use of air-dilution olfactometry might cause less among- and within-individual variability about mood states and PLR parameters, inferencing the association between changes in mood states and PLR parameters due to the robust olfactory stimulation.

In conclusion, our results showed that mental fatigue due to olfactory stimulation might be associated with decreased INIT. Mental fatigue might be a crucial factor in evaluating the association between changes in psychological state and PLR parameters due to olfactory stimulation. The establishment of psychophysical testing enabling objective assessment might improve the ability of diagnosing olfactory impairment. The combination of the T&T olfactometer and autonomic function tests, especially PLR, can be a candidate because of the non-invasiveness. However, as olfactory stimulation can affect psychologically and autonomically with various modalities, further studies should reveal conditions causing psychological changes due to the olfactory stimulation under the co-use of multiple autonomic function tests.

### Supplementary Information

Supplementary Figures.

## Data Availability

The datasets generated during and/or analyzed during the current study are available from the corresponding author on reasonable request.
