# Supplementary material for: Psychological states affecting initial pupil size changes after olfactory stimulation in healthy participants
Source: Sci Rep. 2023 Sep 25;13:16050. doi: 10.1038/s41598-023-43004-1 (PMC10520065; doi:10.1038/s41598-023-43004-1)
Supplement: Supplementary file 1 — Supplementary Figures. [file 41598_2023_43004_MOESM1_ESM.docx]

**
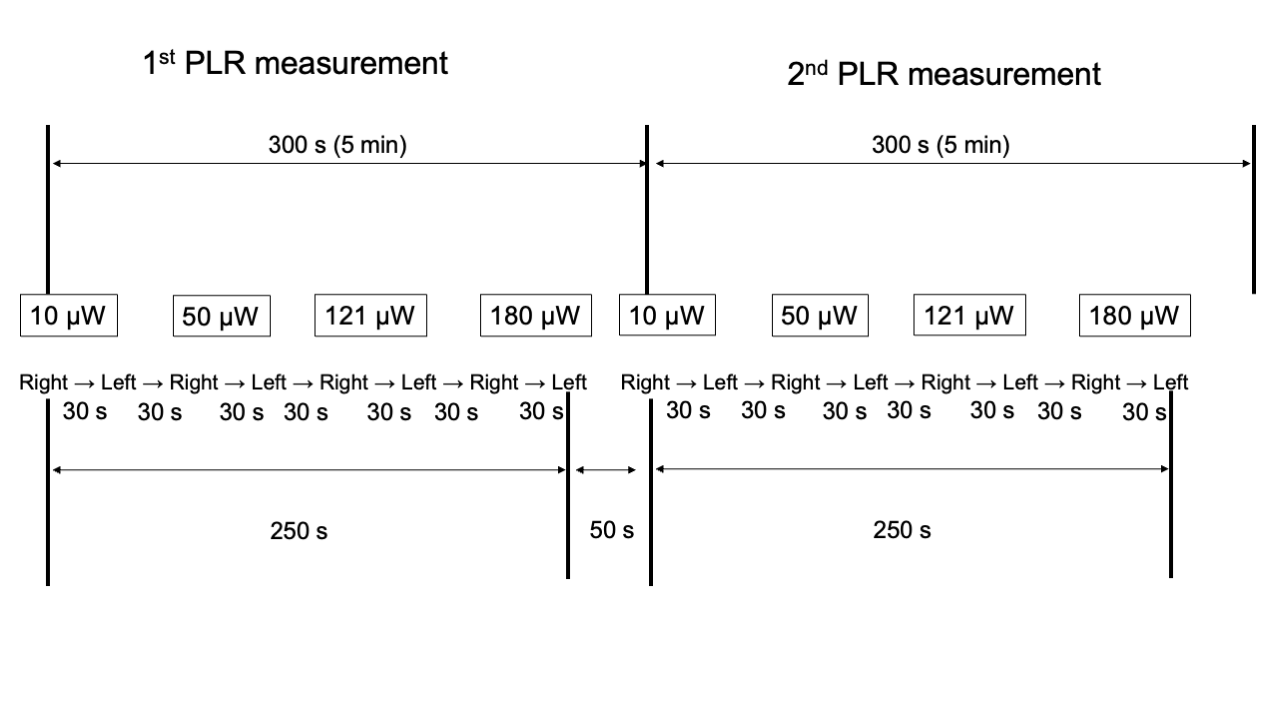
**

**Supplementary Figure 1. Step-up methods for PLR measurement.**

All subjects received 10 μW of light stimulation from the right side to the left side. Afterward, the light stimulus intensities were increased in the following order: (1) 10 μW, (2) 50 μW, (3) 121 μW, and (4) 180 μW. Each interval between PLR measurements was 30 s. The intervals between the end of the first PLR measurement and the first of the 2^nd^ PLR measurement were 50 s. Abbreviation: PLR, pupillary light reflex.

**
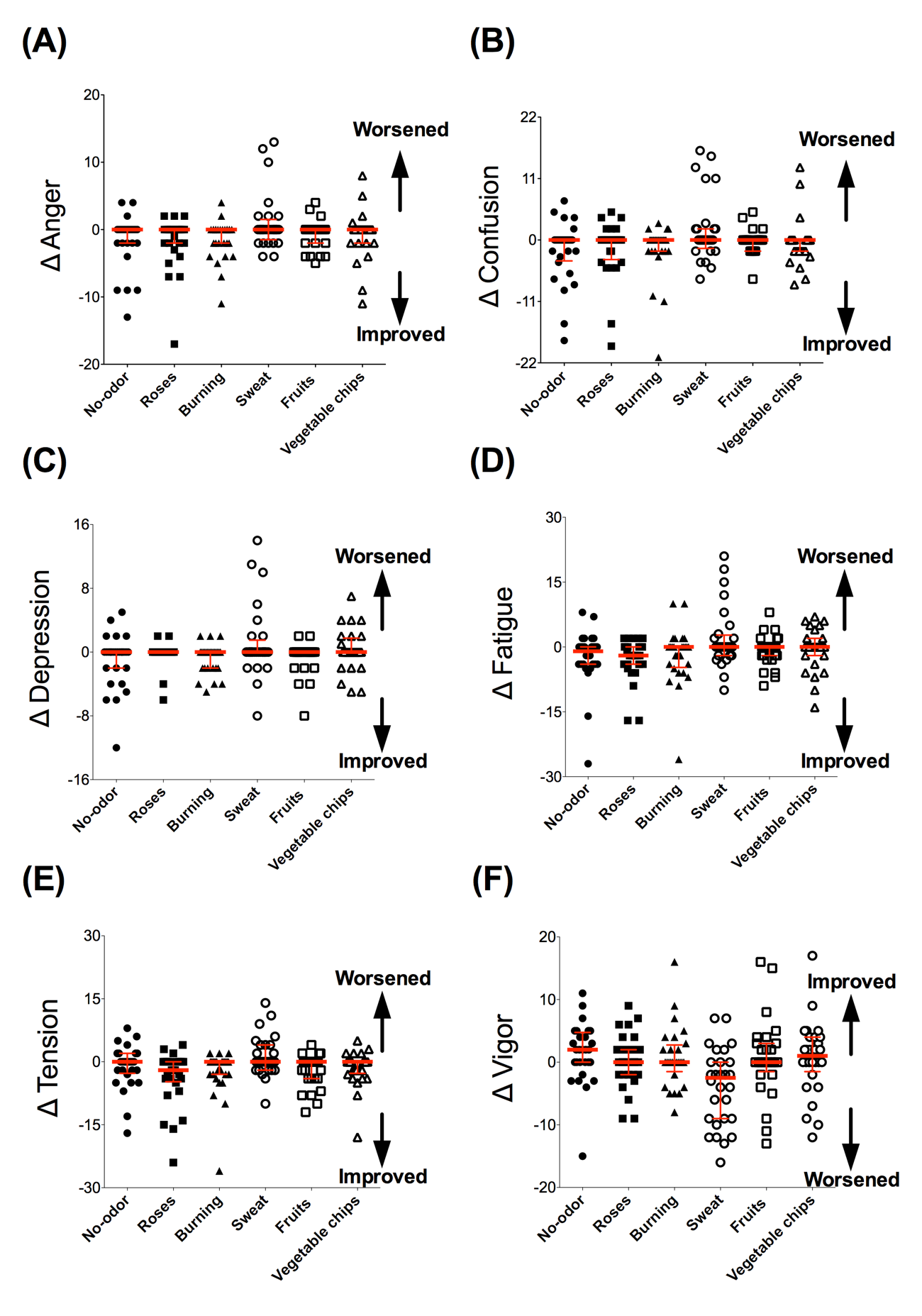
**

**Supplementary Figure 2. Scatter plot showing post-olfactory changes in the mood subscales among six odorants.** For the six odorants, changes in mood subscales calculated by subtracting values at pre-olfactory stimulation from those at post-olfactory stimulation are shown by Δ. Red bars represent the median with the interquartile range. (A) ΔAnger, (B) ΔConfusion, (C) ΔDepression, (D) ΔFatigue, (E) ΔTension, and (F) ΔVigor.

Abbreviations: Anger, anger–hostility; Confusion, confusion–bewilderment; Depression, depression–dejection; Fatigue, fatigue–inertia; Tension, tension–anxiety; Vigor, vigor–activity.


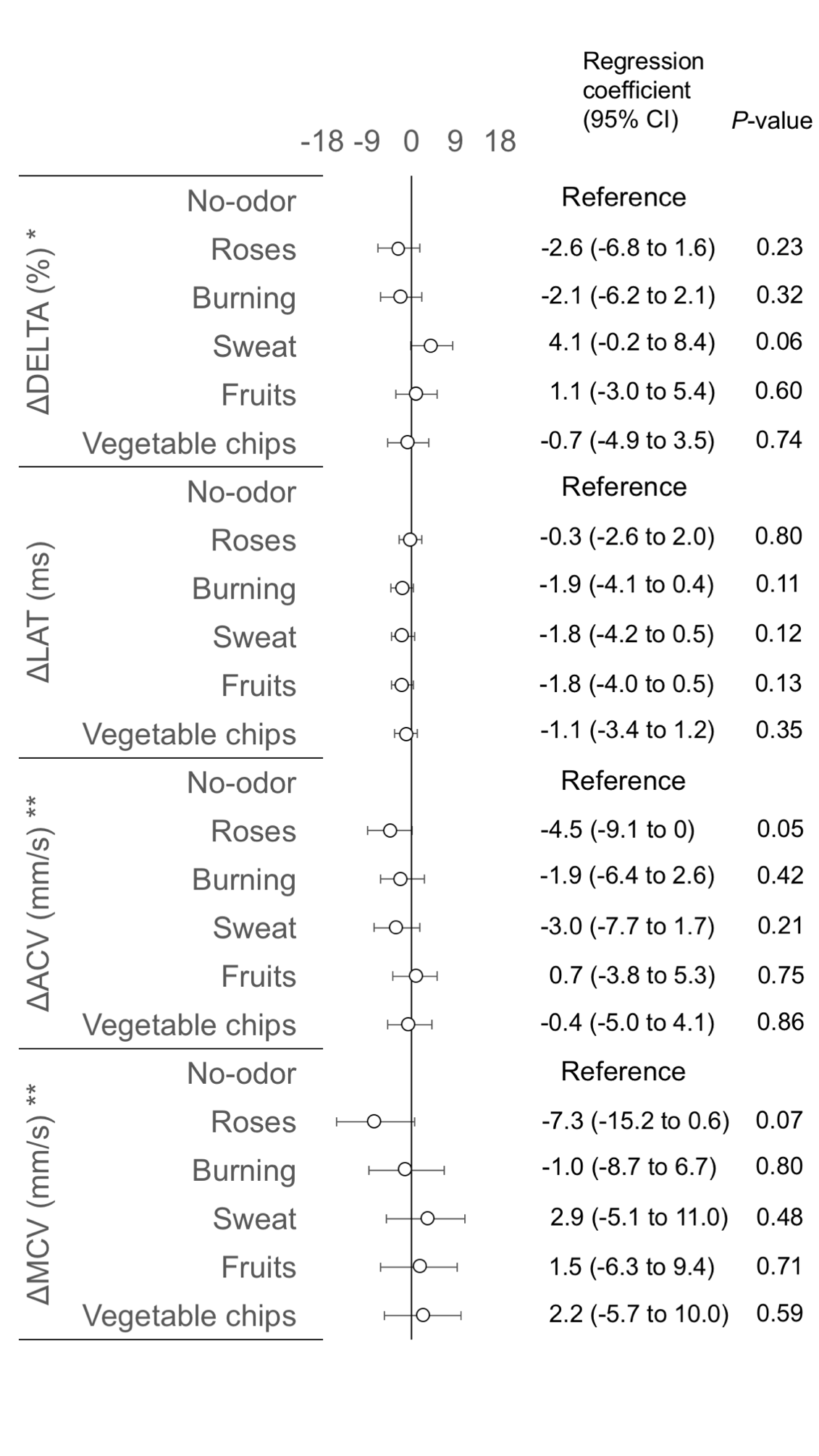


**Supplementary Figure 3. Forest plot of regression coefficients showing changes of DELTA, LAT, ACV, and MCV at the post-olfactory stimulation using six odorants.**

For each of six odorants, changes in PLR parameter are calculated by subtracting values at pre-olfactory stimulation from those at post-olfactory stimulation, shown by Δ. Estimated regression coefficients and p-values calculated by linear mixed-effects models are shown. The no-odor liquid is set as a reference.

Abbreviations: ACV, average constriction velocity; CI, confidence interval; DELTA, constriction ration; LAT, constriction latency; MCV, maximum constriction velocity.
